# Exploring barriers and facilitators of mental health care in Sudurpaschim Province, Nepal: a socioecological qualitative study of patients with depression and anxiety and health care professionals

**DOI:** 10.1186/s12913-025-12983-4

**Published:** 2025-07-01

**Authors:** Gayatri Khanal, Y. Selvamani, Prabhat Sapkota

**Affiliations:** 1 School of Public Health, SRM Institute of Science and Technology (SRMIST), Kattankulathur, Tamil Nadu India; 2 Nisarga Hospital and Research Centre, Dhangadhi, Kailali Nepal

**Keywords:** Barriers, Facilitators, Mental health service, Sudurpaschim Province, Socioecological model

## Abstract

**Background:**

The mental health burden and associated costs are considerable, which is posing challenges for public health delivery systems because of the increased treatment gap, especially in low- and middle-income countries. It is therefore important to investigate the factors influencing access to mental health care from the perspectives of different stakeholders to gain meaningful insights. This study was conducted to assess the existing barriers and facilitators using a socioecological model.

**Methods:**

A qualitative study involving in-depth interviews (IDIs) and focus group discussions (FGDs) at health care facilities was conducted with 24 service users (diagnosed with depression and anxiety) and 21 health care professionals. Thematic analysis was performed and presented using a socioecological model to enhance clarity and impart recommendations.

**Results:**

The results suggest that there are four levels of persisting impediments and facilitators while accessing mental health care in Sudurpaschim Province: (1) Individual level- education, exposure to mass media and women’s empowerment (facilitators) but unawareness and side effects of medication (barriers); (2) Community level: community involvement and awareness (facilitators) while stigma, discrimination, misconceptions, and adverse environments (barriers); (3) Organizational level: psychosocial counselling and effective communication (facilitators) while limited accessibility and availability of MHS (barriers); and (4) Policy level: existing policy and strategies (facilitators) while political indifference, implementation gap and low budget allocation (barriers).

**Conclusions:**

This qualitative study presents a complex, interconnected set of multi-layered barriers and facilitators influencing access to mental health care in Sudurpaschim, Nepal. From the socioecological view, the findings suggest that a comprehensive approach that integrates efforts across all levels are equally essential to effectively address these barriers and facilitators.

**Supplementary Information:**

The online version contains supplementary material available at 10.1186/s12913-025-12983-4.

## Introduction

Across the globe, mental illness is a common issue affecting approximately one out of every eight individuals [1], with the most prevalent conditions being anxiety and depressive disorders [2]. The evidence from the Global Burden of Disease Study indicates that nearly 15% of all diseases or injuries worldwide are caused by mental illness [3]. The associated economic and social costs pose significant challenges to public health system [4]. Moreover, mental illness are responsible for one in six years lived with disability and decreased of life expectancy by 10 to 20 years compared with the average population [5]. There is an enormous treatment gap for most common mental health problems, such as depression and anxiety, which can be treated effectively in low-resource settings through community-based intervention programs and task-sharing approaches [6, 7]. Untreated mental illness continues to have a detrimental impact on global health [8]. This burden, along with the treatment gap, is particularly severe in low-and middle-income countries (LMICs) such as Nepal [9, 10], where an estimated 85–95% of people with mental illness receive either inadequate or no treatment [6, 10]. Thus, there is an urgent need to assess and address the mismatch between the demand for mental health services and the availability and quality of care being provided.

To date, the funding and human resources allocated to MHS in Nepal and in other developing nations remains insufficient, despite evidences identifying these countries as an epicentre of global burden of mental illness [6, 10, 11]. This highlights the urgent need for a comprehensive investigation and a clear understanding of the interconnected barriers and facilitators that influence the delivery of MHS, particularly those living in low-income countries. Numerous studies have illustrated how effectively the present methods of treating mental illness work [6, 7, 12]. Nonetheless, in Nepal, only one in ten patients who need medical assistance seek for professional support [10]. Adults worldwide have expressed reluctance in seeking professional assistance for psychological distress and mental illness for multiple reasons [6], such as stigma [12–14], misconceptions [10, 12, 15], the level of mental knowledge [12, 16–18], long waiting hours [5, 12], a lack of adequate human resources [12], financial scarcity [12], high costs of treatment [19] and distrust between beneficiaries and MHS providers [10, 19].

Mental illnesses such as depression and anxiety are closely linked and can result in numerous adverse outcomes, including poor relationships with family and peers [12, 20], poor job performance [15, 19], and reduced productivity and quality of life [12, 14, 15]. In recent years, several global initiatives including efforts in Nepal have aimed to reduce these negative effects by narrowing the treatment gap for common mental health problems [10]. However, meaningful progress has yet to be achieved [10, 20, 21], largely due a lack of robust evidences to inform and guide for effective strategies [10, 20]. This continues to pose a significant challenge in achieving Target 3.4 of the Sustainable Development Goal, which focuses on reducing premature mortality from non-communicable diseases, including mental health conditions.

The lack of satisfactory progress and the continued negative outcomes underscore the importance of early detection and treatment of mental health illness. This can be achieved by identifying existing barriers, exploring ongoing challenges, and promoting facilitators that support access to mental health services [6, 10, 12]. To date, the underlying causes of treatment gaps for depression and anxiety remains poorly understood due to paucity of evidences [15]. However, a growing body of research indicates that they have a multifactorial etiology, influenced by a combination of personal factors such as education and mental health literacy [16, 22], financial constraints [17, 22] and cultural factors such as religious beliefs and social norms [10, 12, 13].

Bridging this treatment gap requires a thorough understanding of stakeholders’ perceptions in order to encourage individuals to seek professional mental health care [9, 16]. Among the seven provinces of Nepal, Sudurpaschim is one of the least developed and potentially high burden state with mental illness. Hence, the present study was conducted to explore the facilitators and barriers in accessing of MHS from the perspective of both patients’ and health care professionals in Sudurpaschim Province, Nepal.

## Methods

### Study design

A qualitative research method, crucial for exploring issues that are not previously well understood was conducted to reveal the prevailing barriers and facilitators faced by depressive and anxious population while accessing MHS in Sudurpaschim Province, Nepal.

### Study participants, recruitment and study area

**Fig. 1:**
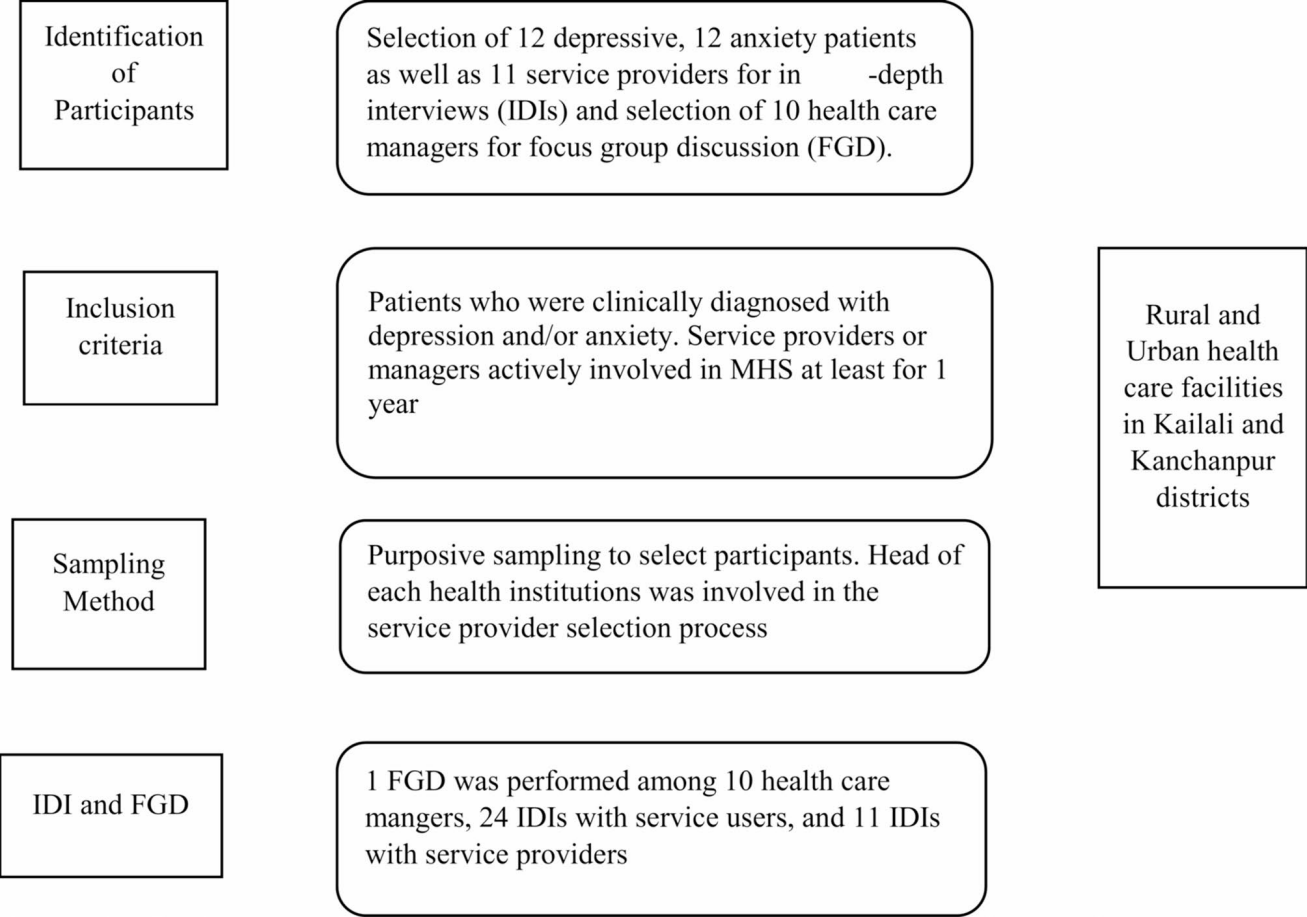
Methodological flowchart

The present study was conducted in rural and urban health care facilities of Kailali and Kanchanpur district of Sudurpaschim Province. Kailali and Kanchanpur were purposively selected, as they are the most populous districts in Sudurpaschim and have diverse ethnic and socio-economic backgrounds. Patients (with anxiety and depression), service providers, and health care mangers were included as study participants. Service providers were selected with the help of the head of health institutions, and patients were recruited from the outpatient department of these health institutions (Supplementary file-1). The inclusion criteria for participation in the study were: (1) individuals clinically diagnosed with depression and/or anxiety, and (2) service providers or managers who had been engaged in MHS profession for at least a year (Fig. 1).

### Sample size and sampling techniques

The total of 45 participants (12- depression, 12-anxiety, 11-service providers, 10- health care mangers) were selected from various hospitals. While conducting 35 IDIs and 1 FGD, a state of theoretical saturation was reached. Hence, further data collection did not yield novel themes or variations, indicating that key concepts had been fully explored and incorporated. The study area and study population were selected using a purposive sampling technique.

### Operational definition

Operational definitions were established for the following terms prior to the commencement of data collection to facilitate participant selection and ensure clarity throughout the study.

#### Health care managers

Individuals with managerial or executive roles, such as medical superintendents, district health officials, public health professionals, and in-charge of primary health care centres or health post.

#### Service providers

Mental health service providers, including Psychiatrists, medical officers, psychiatric nurses, psychosocial counsellors and health assistants (HAs), delivering MHS in public and private health care facilities.

#### Service users

Individuals diagnosed with a psychiatric illness and currently receiving medication for depression and/or anxiety, identified with the assistance of service providers or health care managers.

#### Educated and uneducated

Educated refers to individuals who have received formal schooling. Uneducated refers to individuals who have never attended school.

### Data collection process

Data collection was conducted using local Nepali language by the researchers. IDIs were conducted with patients and service providers, while FGD was held with health care managers from various rural and urban health care facilities of Sudurpaschim Province (Supplementary file-1). The lead investigator initiated the initial interactions with the study participants, and data collection continued until theoretical saturation was reached [23]. IDI and FGD guidelines were developed using broad, and open-ended questions, allowing participants to clearly express their perspectives and feelings in a comprehensive manner. The guidelines for IDI and FGD was developed with the help of previous literatures [14, 15, 17, 22] (Supplementary file-2). Data were recorded using voice recorders and mobile devices, along with field notes by interviewers during the sessions. Interviews conducted in the Nepali language were transcribed and translated into English, with back-translation look over performed by team members (GK and PS) who were fluent in the Nepali language.

### Data analysis

The study employed a thematic analysis approach to identify and explore themes and patterns within the participants’ data, reflecting their perspectives and experiences. All generated themes were mapped onto the socioecological model (SEM) for enhancing clarity [24, 25]. According to the SEM, public health issues, such as barriers in accessing care, arise from the complex interactions of the factors operating at the multiple levels of the model [25]. Using the SEM in qualitative analysis offers a structured yet flexible framework for understanding the complex interaction of barriers and facilitators influencing MHS [24, 25]. Transcripts were meticulously read multiple times before the authors translated the content from Nepali into English language for data analysis. These codes were then categorized and grouped into distinct themes. All transcripts and translations were thoroughly cross-checked by the authors against the original recordings to ensure the accuracy of the information prior to further analysis. The main themes that emerged from the coding framework were identified to represent the narratives of the participants and were subsequently described in detail. To ensure the accuracy of the translated data, the English transcripts were back -translated into Nepalese version. In addition to thematic analysis, the researchers also utilized the R package for qualitative data analysis (RQDA) software to support the analytical process.

### Study rigor

To ensure the credibility of the data, several strategies were employed, including peer debriefing, seeking disconfirmed evidence, and sustained engagement with data. The final categories were determined collaboratively by the full research team. The research methodologies were thoroughly described to enable readers to follow the study process transparently. Finally, confirmability was addressed through the researchers’ effort to disclose all phases of the investigations, allowing readers to explore the data.

## Results

The results section of this study presents the characteristics of the study participants with anxiety and/or depression, service providers, and healthcare managers, and explores the facilitators and barriers to access MHS through a socioecological lens.

Table 1 presents the characteristics of the study participants. A total of 45 individuals participated in this study, consisting of 24 (53.3%) respondents diagnosed with depression and/or anxiety, and 21 (46.7%) health care professionals, including service providers and health care managers. Among the patient group, the majorities were females (66.7%), and most were between 21 and 39 years. Age specifically, 16.7% were ≤ 20 years, 33.3% were 21–29 years, 29.2% were 30–39 years, and 20.8% were 40 years or older, with a mean (Standard Deviation) age of 35.3(4.56) years. Regarding educational status, 58.3% of the patients were educated, while 41.7% were uneducated. In terms of occupation, the highest proportion was involved in agriculture or housework (41.7%), followed by service (25%), students (16.7%), and daily wage basis or foreign employment workers (8.3% each).

**Table 1:** Characteristics of the study participants (*n* = 45)

Respondents/patients with Depression and/or Anxiety	*n* (%) 24(53.3)	Health care professionals (service providers and health care managers)	*n* (%) 21(46.7)
Age (years)			
≤ 20	4(16.7)	≤ 20	0 (0)
>21–29	8(33.3)	21–29	4(19)
30–39	7(29.2)	30–39	10(47.6)
≥ 40	5(20.8)	≥ 40	7(33.4)
Sex			
Male	8 (33.3)	Male	17(81)
Female	16 (66.7)	Female	4(19)
Education			
Educated	14(58.3)	Diploma	5(23.8)
Uneducated	10(41.7)	Undergraduate (UG)	13(61.9)
		Postgraduate (PG)	3(14.3)
Occupation			
Agriculture/housework	10(41.7)	Work experience	
Service	6(25)	Below 5 years	15(71.4)
Daily wages	2(8.3)	5 years or above	6(28.6)
Foreign employment	2(8.3)	Level of health care professions	
Students	4(16.7)	Service providers	11(52.3)
		Health managers	10(47.6)

Among the 21 health care professionals, 81% were male and 19% were female. The age distribution was as follows: 19% were 21–29 years, 47.6% were 30–39 years, and 33.4% were 40 years or older, with a mean (Standard Deviation) age of 33.8(3.32) years. In terms of educational attainment, 23.8% held a diploma degree, 61.9% had an undergraduate degree, and 14.3% completed a postgraduate degree. Most of the professionals (71.4%) had less than five years of work experience, while 28.6% worked for five or more years. The professional categories included 52.3% service providers and 47.6% health managers (Table 1).

**Fig. 2:**
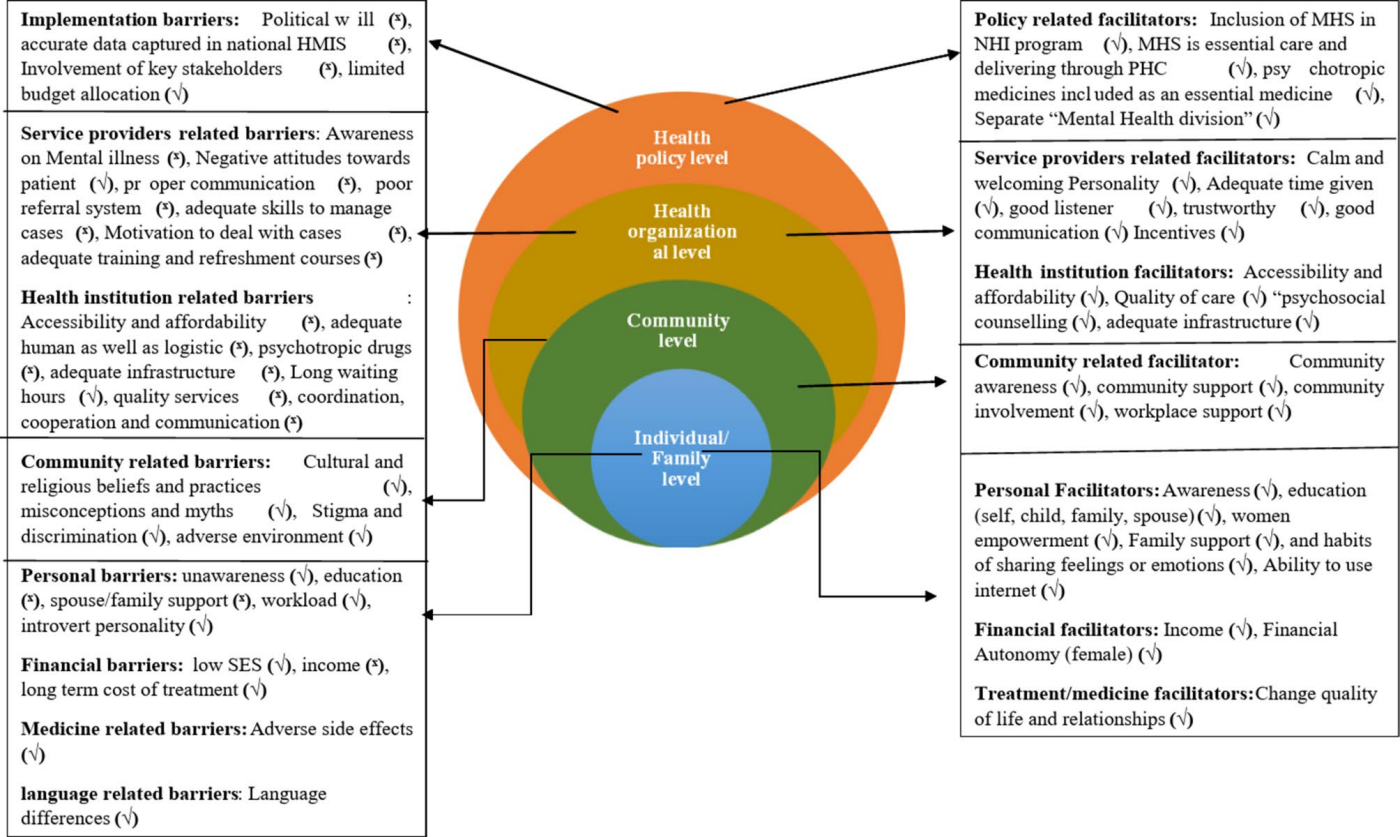
Presentation of barriers and facilitators to access MHS based on socioecological model. Note: **(√)** denotes “Present”, (^**x**^) denotes “Absent”, HMIS: Health Management Information System MHS: Mental Health services PHC: Primary Health Centre

The socioecological model presents four layers of barriers and facilitators of MHS, which are represented in Fig. 2. These levels are the individual level (micro), community level (micro), health organizational level (macro) and policy level (macro).

### Health policy-level barriers and facilitators

At the policy (macro) level, the implementation gaps of policies, strategies, and international commitments were identified as significant challenges in accessing MHS. Participants-particularly those in the FGD emphasized that these gaps are largely driven by a lack of political will, inadequate dissemination of accurate data within the national health information system, scant budget allocation for the mental health, and the tokenistic involvement of critical stakeholders for MHS planning, such as representatives from mental health organizations and patients with mental illness. For example, “The Nepal government has not focused adequately on mental health compared to physical health. *It does not feel good to say but I do not see significant achievement in MHS provision from the past few years*,* even though a mental health strategy exists.” (FGD*,* Health care manger)*.

*“Budget allocation for MHS is scarce*,* and human resource development is insufficient from the government’s side.”(IDI*,* service provider)*.

Despite existing challenges, the Government of Nepal has recently established the “Non-Communicable Disease & Mental Health Section” under the Department of Health Service to facilitate the delivery of MHS across the country. This initiative was perceived by the majority of service providers and healthcare managers as a key motivating factor for the promotion of MHS. Secondly, service providers highlighted the inclusion of various psychotropic medicines in the list of essential drugs available at the PHC level as another important enabling factor. Finally, the National Health Insurance (NHI) program, which covers a limited portion of MHS costs, was also viewed as a motivative measure for increasing MHS utilization.

*“The appreciating part of the Nepal government is incorporating MHS into the NHI. We have observed that patients enrolled in the NHI adhere to treatment more effectively than those who are not.“(FGD*,* Health care manager)*.

*“Now we have a separate mental health section in the Department of Health Services where we can raise and advocate for the challenges encountered in the provision and delivery of MHS.“(FGD*,* Health Care Manger)*.

### Health organizational-level barriers and facilitators

Respondents identified multiple challenges at the organizational level, primarily related to service providers and institutional health-related factors. Firstly, service providers often lacked adequate awareness of mental illness (e.g., depression, anxiety) and had limited training and exposure necessary to manage such cases effectively. Additionally, some respondents perceived negative attitude, discriminatory behavior, and poor communication from health care providers. These factors contributed to irregular treatment and ineffective referrals, further hindering access to mental health services (MHS). Secondly, systemic gaps within the health institutions further exacerbated access to MHS. Both service users and providers reported that limited accessibility, availability, and affordability of health care services had significantly hampered an effective MHS utilization. Resource shortages—such as frequent stockout of psychotropic medications, understaffing leading to longer waiting time, and high patient’s load were seen to reduce the overall quality of care and impair service delivery. Moreover, the lack of private, confidential spaces within health care premises discouraged service users for disclosing and discussing mental health concerns freely.

One patient stated that *“When I entered the* outpatient department (*OPD)*,* there were few other people already sitting beside me*,* which made me uncomfortable sharing all my problems. Hence*,* I only mentioned the symptoms that I thought others wouldn’t pay much attention to. I regret not being able to share that I was having suicidal thoughts too.” (P-1*,* MDD)*.

Patient 10 stated that *“The higher authority does not provide enough psychiatric medicines except for few drugs such as Amitriptyline. Because of this*,* we mostly rely on referral options for other drugs availability to another higher center in Dhangadhi (city).“(P-10*,* Female*,* Depression)*.

A service provider stated that “*Although I took a week-long training for identification and management of mentally ill patients*,* still I fell i am incompetent or unconfident enough to distinguish the type of mental illness. The training felt like watching the movie in one go ‘film here jastaibhayo’. (IDI*,* Service provider)*

Service users also expressed that they felt motivated to seek and continue mental health services (MHS) due to the positive attitude, simple personality, communicative skills, and cozy behaviour of service providers. The qualities of health care service providers such as calmness, hospitality, attentive listening, and allowing sufficient time to express their feelings were highly appreciated by the patients. One patient stated: *“The doctor (Doctor Saab) is excellent. Even though he is an educated and acclaimed person*,* he always smiles*,* speaks kindly*,* and explains things simply the way that I can understand. I have enormous trust in him.*” (P-11, female, (anxiety)).

Patient 6, mentioned, “*My house is hardly 20 min away from this health center*,* so for me*,* it is easy to come for follow-up as per the advice of health workers*.” (P-6, Male, (anxiety)).

Similarly, the utilization of mental health services (MHS) is facilitated by their accessibility and affordability. Service providers emphasized that the availability of psychosocial counselling within healthcare facilities is a crucial factor in delivering effective MHS. Likewise, both service users and providers highlighted that maintaining confidentiality and ensuring privacy within outpatient department (OPD) settings serve as strong motivators for service users to adhere with their treatment plans.

### Community-level barriers and facilitators

Stigma and discrimination emerged as significant impediments to MHS utilization at this micro level. Individuals diagnosed with mental illness were often labeled with derogatory terms such as “mad,” “crazy,” “out of mind,” or “crack.” In some cases, the stigma extended to their families as well, who were referred to as a “mad” or “bad family.” Discriminatory practices included exclusion from family and community gatherings, denial of involvement in household decisions, social avoidance, mocking, teasing, and even physical abuses—such as beating, hitting, throwing objects, or verbal scolding. These behaviors directly discouraged patients from seeking treatment and contributed further to their emotional breakdown and distress.

Respondents also pointed to prevailing misconceptions and cultural myths surrounding mental illness. Many believed that mental health issues were caused by divine punishment, evil spirits, or the result of past misdeeds, and thus could only be addressed through faith-based or traditional healing practices. Mental illness was often viewed as incurable, particularly among young people, who were thought to suffer due to being “weak and helpless.” In Society, marriage was commonly accepted as the only solution for this situation, further deterring individuals from accessing mental health services.

In addition, a few respondents noted that adverse environmental conditions such as emergency situations, the COVID-19 pandemic, natural disasters, and seasonal challenges during winter, the rainy season, or festivals—posed further difficulties in accessing health care.

A patient with *MDD* stated “*My father used to scold me and sometimes beat me too without any reason. He thought I was pretending- doing drama “Natak” -just to get people’s attention.” (P-12*,* Female) A patient (anxiety) stated*,* “I had an extreme fear of dying. Everyone in my family believed that our home God “gharkadevta” was angry because of something we had done. We visited the faith healers*,* and performed several pujas and rituals*,* as instructed.” (P-18*,* Male*,*)*.

*Patient with MDD mentioned*,* “During the coronavirus phase*,* I was unable to take medicine for more than two weeks because of difficulty in travelling outside to purchase medicine. We were not even sure whether we would get medicine even after reaching health post. Because of that*,* my condition became worse.” (P-24*,* Female)*.

A service provider highlighted, *“Most patients tend to skip follow up visits and miss their medication during winter and rainy season.“(Service provider*,* IDI)*.

Support from workplace facilitators, along with growing community awareness and involvement in mental health care, fostered a strong sense of sympathy and encouragement among the respondents. *A male with anxiety stated*,* “I am fortunate to have this manager; he knows what mental illness is. He supports me and gives me permission whenever I need leave for my treatment.” (P-15*,* Male)*.

### Individual-level barriers and facilitators

Lack of awareness regarding the signs and symptoms of mental health conditions, their causes, and available treatments, resulting from limited mental health literacy was identified by nearly all respondents as a major barrier to access mental health care. *“We were not aware of the existence of mental illness. We consider this condition to be a curse/disease of Lords “Devi devatako rog”. My family took me to every traditional healer’s home whom they were aware of. Finally*,* when my family had no options left*,* they took me to the hospital.” (P-10*,* Female)*.

A lack of adequate support from the husband and family members was identified as one of the most significant barriers to access MHS, particularly among females. Heavy household responsibilities, along with introverted personal attributes such tendency to hide illness, hesitant treatment seeking behaviour, and lack of education, were noted as factors that discourage women from utilizing MHS. Participants also highlighted on financial constraints as a major concern. Low socioeconomic status, lack of regular income, and the high cost of long-term treatment including travel and accommodation expenses were seen as major deterrents to seek care. At the personal level, side effects of psychotropic medications, such as dryness of the mouth and throat, drowsiness, disorientation and decreased sexual desire were negatively affecting the treatment adherence. A female patient with MDD told, “*Because of my medicine*,* my sleeping pattern and sexual desire were altered*,* which made my husband unhappy. He became frustrated with the treatment and*,* in furious tone*,* asked me to stop taking it.” (P-7*,* Male) A service provider stated*,* “Most patients visit health facilities only when their condition becomes worse*,* and when no alternative forms of treatments (e.g.*,* Jharfuk) are effective and available.”*

On the other hand, many personal characteristics promote the utilization of MHS, such as awareness of mental illness, family education and support, women’s empowerment and financial status. A patient (major depressive disorder), expressed, *“My daughter is working as a clinical nurse. She is the one who convinced me to visit a psychiatric doctor after seeing my behavioural changes such as looking unhappy*,* staying alone and avoiding social programs.”* (P-1, Male).

A good socioeconomic status, including personal/family income and financial autonomy, especially for females, was commonly witnessed as a facilitator by respondents at the individual level. *“My husband is receiving his pension each month*,* and two of my children are working in a foreign country. Everyone in my family encouraged me to obtain the best possible treatment and not to worry about money.”*(P-2, Female).

Likewise, noticeable improvements in quality of life and interpersonal relationships following treatment or medication also play crucial role in promoting compliance. *A Patient with MDD expressed*,* “After this treatment*,* I feel like I have been offered a new life (arko juni paye jasto bhayo). I don’t even regret selling my ornaments to pay for it.” (P-14*,* Female)*.

A service provider mentioned that *“female patients tend to be more consistent and compliable with treatment when they notice improvements in their condition and relationships.” (Service provider*,* IDI)*.

## Discussion

The purpose of this paper is to explore the perspective of individuals (with anxiety and depression) and health care professionals (service providers and health care managers) regarding the obstacles and facilitators experienced while receiving MHS. Because the socioecological model sheds light on the intricacies and difficulties faced by healthcare workers and service users on a daily basis, it provides a suitable framework for investigating the subject. The study examines several barriers and facilitators at different scales, from micro to macro, and provides recommendations considering the interplay and interconnection of these levels.

The study main findings are echoed by previous researches from various low- and middle-income countries (LMICs) on obstacles preventing mental treatment [14, 15, 26–41] and opportunities promoting for utilization of MHS [14–18, 26, 27, 28, 33, 34, 37, 42]. The application of the socioecological model (SEM) helped in identifying key barriers systematically and enabling at multiple levels to influence and ensure the delivery of mental health screening and treatment [28–30]. The findings of these studies suggest that, to provide optimum care for patients with depression and anxiety, the supply side should modify or update programs/policies, expand funding as well as improve patient accessibility to MHS [5–7]. In addition, there is a pressing need to disseminate accurate information on MHS through community-led interventions and awareness campaigns. These initiatives are vital to address and reduce stigma, debunk myths, and correct misconceptions surrounding mental illness [5, 26, 36]. Awareness campaigns are particularly effective in enhancing access to mental health care by reducing stigma, increasing mental health literacy, and encouraging help-seeking behaviour. They often utilize diverse platforms—such as social media, community events, and educational programs—to disseminate information and normalize conversations around mental health [33].

The negative perceptions of mental illness, especially stigma and discrimination significantly hinder the utilization of MHS, as reported by the majority of our service receivers. This aligns with several prior studies that emphasized the impact of stigma on help-seeking behaviour among individual with mental illness [13–20, 34, 35, 38–41]. In particular, patients being labelled as “committed a major sin” adds to the burden of discrimination and further discourages treatment-seeking attitude. Systematic reviews by Smith et al. in the UK and DeSa et al. in high-income countries such as revealed that stigma and discrimination are complex barriers connected from the micro to the macro level of treatment-seeking pathways [42, 43]. A similar comparison was observed in various studies, considering that awareness of mental illness is a major facilitating factor for service utilization [12–16, 26, 28, 37, 42]. Several studies linked with specific cultural norms (performing rituals for Devidevata) and traditional treatment modalities, are against mental illness screening and treatment, which were found as impediments as per the studies [10, 14–17, 22, 27, 33, 39–42].

These results suggest that increasing mental health literacy and awareness to reach its zenith is still a top priority and could be a encouraging factor for people to seek treatment easily and quickly. Mass media can be an excellent platform for enhancing mental health education, leading to increased utilization of existing MHS [7, 42, 43]. Encouraging and enhancing community involvement and community organization on mental illness is imperative for sustainable mental health care utilization [5–7, 43].

At the health organizational (macro) level, one of the significant barriers to MHS utilization is lack of adequate training and refresher courses for service providers, resulting in incompetent care for individuals with mental illness [26, 35]. Furthermore, owing to difficult access, people need to travel miles and expend substantial money to reach health institutions, which was mentioned similarly by several studies conducted in different settings [27, 34, 37–40]. An enlarged qualitative study synthesized findings indicating that the unavailability of health care services [20, 34, 38], a shortage of human resources [26–28, 30, 34–41], and an inconsistent and minimal supply of psychiatric medicines [5–8, 10, 14, 15, 27–39, 42, 43] are by far significant barriers to access MHS. Political motivation is limited to the provision and expansion of MHS, which is consistent with the findings generated through other scientific evidences [6, 10, 12, 14, 44]. The absence of political will at the macro level negatively impacts all other levels of service delivery, leading to reduced engagement and demand from service users [44]. This study underscores the value of a hybrid, multilevel approach that advocates a reform on systemic policy from the grassroot level. Specifically, it highlights the need for tailored community sensitization initiatives, mobilization of local support systems, training for targeted health workers, and provision of a reliable supply chain for psychotropic medications [12, 42–44].

The shortage of human resources indirectly increases the workload for service providers and long waiting hours for patients [13–15, 20, 36]. Provision of an adequate number of trained dedicated service providers, isolated confidential spaces for counselling, provision of incentives and motivational benefits to service providers, strengthening the supply chain for medicine management, enhancing the supervision system and referral system, and keeping appropriate records in the national health information system would be the prime facilitating factors for treatment adherence, which needs to be incorporated into the mental health delivery system [12, 14, 43].

### Strengths and limitations

This paper comprehensively identified the barriers and facilitators influencing access to MHS, framing them through a socioecological perspective which is an important strength of this study. Additionally, unlike earlier researches which primarily focused on individuals with depression and anxiety, our study included a diverse group of service providers and health care mangers as well offering a more holistic and realistic understanding of the challenges and mediators of MHS. Further stating the strength, it explored not only barriers but also facilitators, which is essential for clear understanding the contributing factors that affect mental health service utilization [17]. We used purposive sampling in this study, which may have led to the inclusion of participants who were more outspoken and approachable, potentially introducing a selection bias. Furthermore, recruitment was limited to individuals who were already engaged in providing MHS. This may have excluded certain hidden barriers and facilitators experienced by those who have not sought help or visited mental health premises, leading to an incomplete picture of the challenges in seeking care. This study was conducted solely in the Nepali language which although is widely spoken, may not be the first language for everyone in the Far-Western region. This may have imposed certain limitations on participation or influenced responses, thereby potentially affecting the inclusivity and representativeness of the findings.

At the end, the study focused exclusively on patients with depression and anxiety in Sudurpaschim province. This may limit the generalizability of the findings to other provinces and individuals with other mental illness.

## Conclusions

This qualitative study laid out a complex and interlinked framework based on the socioecological model, highlighting multilevel barriers and facilitators in accessing mental health services (MHS). The findings reveal a framework of persisting challenges and enablers at four sectors starting from the individual/family, community, organizational, and policy level. At the individual level, factors such as education, media exposure, and women’s empowerment facilitate, while lack of awareness and concerns over medication side effects act as barriers to MHS utilization. At the community level, stigma, discrimination, and misconceptions hinder access to MHS, despite positive impact of community involvement and awareness programs. At the organizational level, psychosocial counselling and effective communication support access to the care, yet a lack of available, accessible and affordable mental health services remains a major obstacle. Lastly, at the public policy level, existing strategies and policies provide a foundation for mental health care, but gaps in political interest, implementation and limited financial resources constrain their effectiveness.

To enhance access to care, this study suggests that evidence-based interventions targeting specific factors (barriers and facilitators) at each level would be crucial. A multi-faceted approach is essential, involving policy advocacy, workforce expansion, community-led interventions, and digital media campaigns. Additionally, improving mental health literacy, increasing budget allocations, and expanding resources and services are critical steps toward addressing the challenges faced by individuals in Sudurpaschim Province, Nepal. Future research should aim to provide a deeper comparative analysis, including patients who are not only engaged in mental health care, comprehensively determine both barriers and facilitators across multiple levels, and provide effective, context-specific interventions.

## Supplementary Information

Supplementary Material 1.

Supplementary Material 2.

## Data Availability

The datasets used and/or analyzed during the current study are available from the corresponding authors (Gayatri Khanal and Dr. Y. Selvamani) on reasonable request.
