# Supplementary material for: Exploring barriers and facilitators of mental health care in Sudurpaschim Province, Nepal: a socioecological qualitative study of patients with depression and anxiety and health care professionals
Source: BMC Health Serv Res. 2025 Jul 1;25:855. doi: 10.1186/s12913-025-12983-4 (PMC12219968; doi:10.1186/s12913-025-12983-4)
Supplement: Supplementary file 1 — Supplementary Material 1. [file 12913_2025_12983_MOESM1_ESM.docx]

**List of the selected hospital and PHC from urban and rural areas of Kailali and Kanchanpur district**

| **Kailali** | | **Kanchanpur** | |
| --- | --- | --- | --- |
| Urban | Rural | Urban | Rural |
| Nova (SP-1, HCM-1, Patient-4 | Joshipur PHCc (SP-1, HCM-1, Patient-2 | Mahakali Hospital (SP-1, HCM-1, Patient-3  ) | Belauri PHC (SP-1, HCM-2, patient -3 |
| Nisarga (SP-3, HCM-1, Patient-6 | Bhajani PHCC (SP-1, HCM-1, Patient-1) | Mantra Hospital (SP-1, HCM- 1Patient-3) | Dodhara PHC (SP-1, HCM-1, Patient-2 |
| Seti provincial hospital (HCM-1) |  |  | |

**Total Patient-24, SP-11, HCM-10**

**Note: SP (Service Providers), HCM (Health Care Manager)**

**Mental Health services provided by selected hospital)**

Nova, Nisarga, Mahakali and Mantra – Out Patient Department (OPD) services for all type of mental illness, Inpatient department (IPD) (But separate psychiatry ward available) as well as counselling services.

Seti provincial hospital: No OPD, IPD services though it is a provincial hospital

Joshipur, Bhajani, Belauri and Dodhara PHC: OPD services/basic mental health services only for most common illness (Anxiety, Depression, Suicidality, conversion, psychoses, epilepsy and alcohol use disorder)
