# Supplementary material for: Exploring barriers and facilitators of mental health care in Sudurpaschim Province, Nepal: a socioecological qualitative study of patients with depression and anxiety and health care professionals
Source: BMC Health Serv Res. 2025 Jul 1;25:855. doi: 10.1186/s12913-025-12983-4 (PMC12219968; doi:10.1186/s12913-025-12983-4)
Supplement: Supplementary file 2 — Supplementary Material 2. [file 12913_2025_12983_MOESM2_ESM.docx]

1. **Semi- structure in-depth interview guide for patients with depression and anxiety**

Name:

Age:

Sex:

Caste:

Religion:

Occupation:

Type of the problem:

To meet the research objectives, the researchers developed an interview guide informed by prior studies, particularly those conducted in Nepal. The following questions will be posed to participants (experiencing depression or anxiety), with additional questions included as needed based on their responses and perspectives:

1. From your experience as a depressive/anxiety patient under mental health services at this hospital, what do you feel when you realize that you suffer from anxiety/depression?
2. Reflecting on your experiences with health care, what personal obstacles have prevented you from seeking treatment at a health facility? Similarly, what are the personal reasons that motivating factors encourage you to seek treatment?
3. In your opinion, what institutional factors within the health care system discourage or encourage you from using mental health services? For instance, how do the behavior of health care providers or extended waiting times affect your experience?
4. How do cultural or religious beliefs influence your use of mental health services at this hospital? If you do not seek care at the hospital, where else do you go for support?
5. What environmental challenges and opportunities do you face that prevent/encourage you from accessing mental health care at hospitals?
6. How do you, your family, and your community perceive your mental health condition?
7. What financial difficulties limit your ability to access mental health services at hospitals?
8. From your perspective, what personal changes or situations, compared to the past, motivate you or others in your community to seek mental health services? What are these factors?
9. Based on your experiences, what institutional aspects of this hospital encourage you to use its mental health services?
10. If there are other barriers or facilitators affecting your use of mental health services, please share your perspective.
11. **Semi- structure in-depth interview guide for Health care workers**

Name:

Age:

Sex:

Caste:

Religion:

Occupation:

Experience:

1. From your experience, when a patient with depression or anxiety comes for treatment at this hospital, what actions do they take or how do they feel when they realize they are suffering from anxiety or depression?
2. From your experience as a mental health service provider at this hospital, what personal barriers have you observed that prevent patients with depression or anxiety from seeking care at a health facility? Or the personal factors which motivates them to seek treatment?
3. Based on your experiences, what organizational barriers have you encountered that prevent patients from seeking health care at this facility? Alternatively, what challenges do you face in delivering mental health care at your institution? What are the facilitating factors in organizational level that motivates health care workers or patients from giving or receiving care?
4. What social influences or obstacles hinder the integration of mental health care at this hospital?
5. From your perspective, are there any factors or circumstances, compared to the past, that motivate people to utilize mental health care? If so, what are they?
6. What cultural or religious factors limit access to or the use of mental health care services at this hospital? When people do not seek care here, where else do they go for mental health services?
7. What environmental challenges do you face that make it difficult to seek or provide mental health care services at hospitals?
8. What are the prevailing perceptions and attitudes of family and community members toward mental disorders?
9. If you believe there are other barriers or facilitators affecting mental health service utilization, please share your perspective.
10. **Content for FGD Discussion for Health care managers**
    1. From your experience as a health care manager, what personal barriers have you observed that prevent patients with depression or anxiety from seeking care at a health facility?
    2. Based on your experiences, what organizational barriers have you encountered that prevent patients from seeking health care at this facility? Alternatively, what challenges do you face in managing mental health services at your localities?
    3. What social influences or obstacles hinder the integration of mental health care in the grassroot level health care delivery?
    4. From your perspective, are there any factors or circumstances, compared to the past, that motivate people to utilize mental health care? If so, what are they?
    5. What cultural or religious factors limit access to or the use of mental health care services at this hospital? When people do not seek care here, where else do they go for mental health services?
    6. What environmental /policy related challenges do you face that make it difficult to provide mental health care services at hospitals? Or factors motivate mental health services in your localities?
    7. What are the prevailing perceptions and attitudes of family and community members toward mental disorders?
    8. As a health care manager, what are the challenges did you face to delivery effective mental health services in the health post, primary Health Care Centres and how can we overcome this?
    9. If you believe there are other barriers or facilitators affecting mental health service utilization from your, health care workers and patients’ side, please share your perspective.

**Thank you**
